# Supplementary material for: HIVprotI: an integrated web based platform for prediction and design of HIV proteins inhibitors
Source: J Cheminform. 2018 Mar 9;10:12. doi: 10.1186/s13321-018-0266-y (PMC5845081; doi:10.1186/s13321-018-0266-y)
Supplement: Supplementary file 2 — Additional file 2. Source code of HIVProtI web server. [file 13321_2018_266_MOESM2_ESM.zip › HIVprotI_Source-code/hivprotI/weka-3-6-6/doc/weka/classifiers/bayes/net/MarginCalculator.html]

MarginCalculator


---


|  |  |  |
| --- | --- | --- |
| **Overview** \| **Package** \| **Class** \| **Tree** \| **Deprecated** \| **Index** \| **Help** \| **Weka's home** | |  |
| **PREV CLASS**   **NEXT CLASS** | **FRAMES**    **NO FRAMES**     **All Classes** |  |
| SUMMARY: NESTED \| FIELD \| CONSTR \| METHOD | DETAIL: FIELD \| CONSTR \| METHOD |  |


---


## weka.classifiers.bayes.net Class MarginCalculator

```
java.lang.Object
  weka.classifiers.bayes.net.MarginCalculator
```

**All Implemented Interfaces:**: java.io.Serializable, RevisionHandler

---

``` public class MarginCalculator extends java.lang.Object implements java.io.Serializable, RevisionHandler ```

**See Also:**: Serialized Form

---

| **Nested Class Summary** | |
| --- | --- |
| `class` | `MarginCalculator.JunctionTreeNode` |
| `class` | `MarginCalculator.JunctionTreeSeparator` |


| **Field Summary** | |
| --- | --- |
| `MarginCalculator.JunctionTreeNode` | `m_root` |


**Constructor Summary**

`MarginCalculator()`


| **Method Summary** | |
| --- | --- |
| `void` | `calcFullMargins(BayesNet bayesNet)` |
| `void` | `calcMargins(BayesNet bayesNet)`             Calc marginal distributions of nodes in Bayesian network Note that a connected network is assumed. |
| `boolean[][]` | `fillIn(int[] order, boolean[][] bAdjacencyMatrix)`             Apply Tarjan and Yannakakis (1984) fill in algorithm for graph triangulation. |
| `double[]` | `getMargin(int iNode)` |
| `int` | `getNode(java.lang.String sNodeName)` |
| `java.lang.String` | `getRevision()`             Returns the revision string. |
| `static void` | `main(java.lang.String[] args)` |
| `boolean[][]` | `moralize(BayesNet bayesNet)`             moralize DAG and calculate adjacency matrix representation for a Bayes Network, effecively converting the directed acyclic graph to an undirected graph. |
| `void` | `process(boolean[][] bAdjacencyMatrix, BayesNet bayesNet)` |
| `void` | `setEvidence(int nNode, int iValue)` |
| `java.lang.String` | `toString()` |
| `java.lang.String` | `toXMLBIF03()` |

**Methods inherited from class java.lang.Object**

`equals, getClass, hashCode, notify, notifyAll, wait, wait, wait`

**Field Detail**

### m\_root

```
public MarginCalculator.JunctionTreeNode m_root
```


**Constructor Detail**

### MarginCalculator

```
public MarginCalculator()
```


**Method Detail**

### getNode

```
public int getNode(java.lang.String sNodeName)
```

---


### toXMLBIF03

```
public java.lang.String toXMLBIF03()
```

---


### calcMargins

```
public void calcMargins(BayesNet bayesNet)
                 throws java.lang.Exception
```

:   Calc marginal distributions of nodes in Bayesian network
    Note that a connected network is assumed.
    Unconnected networks may give unexpected results.

    :   **Parameters:**: `bayesNet` - **Throws:**: `java.lang.Exception`

---


### calcFullMargins

```
public void calcFullMargins(BayesNet bayesNet)
                     throws java.lang.Exception
```

:   **Throws:**: `java.lang.Exception`

---


### process

```
public void process(boolean[][] bAdjacencyMatrix,
                    BayesNet bayesNet)
             throws java.lang.Exception
```

:   **Throws:**: `java.lang.Exception`

---


### moralize

```
public boolean[][] moralize(BayesNet bayesNet)
```

:   moralize DAG and calculate
    adjacency matrix representation for a Bayes Network, effecively
    converting the directed acyclic graph to an undirected graph.

    :   **Parameters:**: `bayesNet:` - Bayes Network to process **Returns:**: adjacencies in boolean matrix format

---


### fillIn

```
public boolean[][] fillIn(int[] order,
                          boolean[][] bAdjacencyMatrix)
```

:   Apply Tarjan and Yannakakis (1984) fill in algorithm for graph
    triangulation. In reverse order, insert edges between any non-adjacent
    neighbors that are lower numbered in the ordering.
    Side effect: input matrix is used as output

    :   **Parameters:**: `order:` - node ordering: `bAdjacencyMatrix:` - boolean matrix representing the graph **Returns:**: boolean matrix representing the graph with fill ins

---


### setEvidence

```
public void setEvidence(int nNode,
                        int iValue)
                 throws java.lang.Exception
```

:   **Throws:**: `java.lang.Exception`

---


### toString

```
public java.lang.String toString()
```

:   **Overrides:**: `toString` in class `java.lang.Object`

---


### getMargin

```
public double[] getMargin(int iNode)
```

---


### getRevision

```
public java.lang.String getRevision()
```

:   Returns the revision string.

    :   **Specified by:**: `getRevision` in interface `RevisionHandler`
    :   **Returns:**: the revision

---


### main

```
public static void main(java.lang.String[] args)
```


---


|  |  |  |
| --- | --- | --- |
| **Overview** \| **Package** \| **Class** \| **Tree** \| **Deprecated** \| **Index** \| **Help** \| **Weka's home** | |  |
| **PREV CLASS**   **NEXT CLASS** | **FRAMES**    **NO FRAMES**     **All Classes** |  |
| SUMMARY: NESTED \| FIELD \| CONSTR \| METHOD | DETAIL: FIELD \| CONSTR \| METHOD |  |


---
